# Full Dimensional Potential Energy Function and Calculation of State-Specific Properties of the CO+N_2_ Inelastic Processes Within an Open Molecular Science Cloud Perspective

**DOI:** 10.3389/fchem.2019.00309

**Published:** 2019-05-22

**Authors:** Andrea Lombardi, Fernando Pirani, Massimiliano Bartolomei, Cecilia Coletti, Antonio Laganà

**Affiliations:** ^1^Dipartimento di Chimica, Biologia e Biotecnologie, Università di Perugia, Perugia, Italy; ^2^Consortium for Computational Molecular and Materials Sciences (CMS)^2^, Perugia, Italy; ^3^Instituto de Física Fundamental, Consejo Superior de Investigaciones Científicas, Madrid, Spain; ^4^Dipartimento di Farmacia, Università “G. d'Annunzio” Chieti-Pescara, Chieti, Italy; ^5^CNR ISTM-UOS Perugia, Perugia, Italy; ^6^Master-UP srl, Perugia, Italy

**Keywords:** intermolecular interactions, molecular energy transfer, rate constants, molecular beams, astrochemistry, carbon monoxide, planetary atmospheres

## Abstract

A full dimensional Potential Energy Surface (PES) of the CO + N_2_ system has been generated by extending an approach already reported in the literature and applied to N_2_-N_2_ (Cappelletti et al., 2008), CO_2_-CO_2_ (Bartolomei et al., 2012), and CO_2_-N_2_ (Lombardi et al., 2016b) systems. The generation procedure leverages at the same time experimental measurements and high-level ab initio electronic structure calculations. The procedure adopts an analytic formulation of the PES accounting for the dependence of the electrostatic and non-electrostatic components of the intermolecular interaction on the deformation of the monomers. In particular, the CO and N_2_ molecular multipole moments and electronic polarizabilities, the basic physical properties controlling the behavior at intermediate and long-range distances of the interaction components, were made to depend on relevant internal coordinates. The formulated PES exhibits substantial advantages when used for structural and dynamical calculations. This makes it also well suited for reuse in Open Molecular Science Cloud services.

## 1. Introduction

The presence of significant traces of CO in gaseous systems in which molecular nitrogen N_2_ is an abundant component is a frequent situation in astrochemistry, plasma chemistry and combustion. Besides obvious implications for combustion and CO_2_ plasmas, CO plays an important role in planetary atmospheric chemistry (He et al., 2017), and it has been detected in Titan, Triton, and Pluto as a stable minor constituent (Lellouch et al., 2010), contributing to the energy balance of the planet and the chemistry of small organic molecule formation. For example, recent studies consider the contribution of CO to the formation and role of atmospheric hazes present in a number of solar system and exoplanetary atmospheres (He et al., 2017) and, particularly, hydrocarbon hazes, which have substantial radiative heating and cooling effects in such atmospheres, as observed for Saturn's moon Titan (Tomasko et al., 2008; He et al., 2017; Hörst et al., 2018) and for Jupiter's stratosphere (Zhang et al., 2015) (not to mention the role of CO in the Earth's atmosphere chemistry). Organic hazes are particularly interesting due to astrobiological implications, such as the potential for Titan's atmosphere to contain the building blocks of life (Hörst, 2012; Fabiano et al., 2017). It was recently shown in a series of atmosphere simulation experiments using gas mixtures of CO, CH_4_, and N_2_ (Hörst et al., 2018) that the inclusion of CO has a dramatic effect on the gas phase chemistry as well as on the density and composition of the solid material that is formed. In this respect, CO can actually be seen as the smallest molecule that could serve as a source of oxygen and carbon in space and planetary atmospheres.

The knowledge of the intermolecular interactions involving carbon monoxide and nitrogen (as well as methane) is generally required in the above mentioned fields to correctly assess their role in the chemical kinetics of gaseous environments. A fundamental issue is the accurate representation of the set of energy transfer processes involving CO in the various kinetic models used to study the gaseous mixtures.

Collisions between molecules promote transfer of energy amongst translational, rotational and vibrational degrees of freedom, determining the molecular state population. Dynamics simulations based upon quantum, classical and semiclassical scattering calculations permit one to obtain cross sections and thermal or state-to-state rate coefficients (Lombardi et al., 2013, 2015, 2016a; Celiberto et al., 2016). The resulting data, refined and complemented by experimental data (whenever available) can be used to set up relevant parts of kinetic models (Kustova and Kremer, 2014), routinely used in many research fields to simulate complex environments such as combustion mixtures, plasmas, atmospheres and molecular clouds in in the interstellar medium (Bacmann et al., 2002). The energy transfer processes in each collision event, being generally strongly state-specific, are sensitive to the initial quantum states of the involved molecules. Therefore, energy exchange kinetics is better accounted for by appropriate sets of state-to-state rate coefficients, rather than averaged. Indeed, state-to-state coefficients are entirely necessary when non-equilibrium conditions prevail. In those cases, the collection of reliable energy transfer cross sections and rate coefficients is the true accuracy-determining step of the modeling (Kustova and Nagnibeda, 2012; Kustova and Kremer, 2014).

In turn, the accuracy of whatever rate coefficient obtained by calculations depends on the realism of the underlying dynamics simulations, which essentially means a very accurate description of the intermolecular interactions, occurring at long- and mid-range distances, with the energy exchanges being to a large extent due to such forces. Finally, the need for accuracy has to be contrasted with the computational demand of campaigns of massive state-to-state coefficient calculations. In this respect, flexible and easy to reuse potential energy surface formulations play an important role in the cooperative assemblage of simulations of chemical processes based on atomistic approaches. However, most of the structural and dynamics data available in popular data banks, of interest in several fields, including astrochemistry, combustion and plasmas, lack solid validation and are sometimes even totally arbitrary. A typical weakness of the mentioned data consists of the fact that even those originating from high-level ab initio calculations might be lacking sufficient accuracy in the long-range region. For this purpose, we currently check the suitability of the proposed PESs for carrying out calculations of the detailed probabilities, cross sections and rate coefficients for inelastic processes of the title systems.

Our work, however, due to the general high demand of computational resources that kinetics and dynamics simulations generate, has also the broader prospective aim of building MOlecular Simulator Enabled Cloud Services (MOSEX) (Vitillaro and Laganà, 2018) meant to provide the Molecular Science Community with a Cloud service (Laganà et al., 2018b) by offering the provision of accurate estimates of molecular properties generated in (often collaborative) experiments and validated by high-level theory (also often collaborative) simulations.

Accordingly, the purpose of the present work is threefold. First is to illustrate the development of an accurate full dimensional PES describing the intermolecular interactions of CO-N_2_ systems. This is done by extending a well established semiempirical approach, based on a bond-bond description of the interactions (see e.g., Cappelletti et al., 2008; Faginas Lago et al., 2013 and references therein).

Second is to discuss a dynamical preliminary validation of the proposed PES through application to the case of the basic Vibration-Vibration (VV) energy exchange processes of CO in nitrogen-containing mixtures: CO(1)+N_2_(0) → CO(0)+N_2_(1) and N_2_(1)+CO_2_(0) → N_2_(0)+CO_2_(1).

Third is to report on the progress made in providing calculated scattering properties as a service to the community using the so-called Grid Empowered Molecular Simulator (GEMS) (Laganà et al., 2010; Manuali et al., 2010; Rampino et al., 2012a) through the activities of the Virtual Organization (VO) COMPCHEM (Laganà et al., 2010) first and of the Chemistry Molecular and Materials Science and Technologies (CMMST) Virtual Research Community (VRC) (Laganà, 2012) later.

The paper is therefore organized as follows. Section 2 is devoted to the description, formulation and optimization of the proposed CO+N_2_ PES. Section 3 is devoted to illustrating the quantum-classical calculations and related results. Section 4 is devoted to illustrating the implemented Open Molecular Science Cloud prototype.

## 2. The Potential Energy Surface

In this section we discuss the formulation of the PES of the CO-N_2_ system used for the calculations and briefly illustrate as well its optimization and extension to flexible monomers. The approach we use is based on the so called bond-bond method (Cappelletti et al., 2008), a valuable feature of which is the choice of expressing the potential function parameters in terms of bond properties and parameters characterizing the internal molecular structure, such as charge distributions and polarizabilities. Since the energy functions depend on parameters having a well-defined physical meaning, these are portable in different molecular environments as building blocks of force fields of, in principle, whatever complexity, as testified by the application of the method to a variety of systems (see e.g., Albertí et al., 2011; Albertí and Faginas Lago, 2012; Bartolomei et al., 2012; Lombardi et al., 2012, 2016b; Falcinelli et al., 2013; Pacifici et al., 2013; Faginas-Lago et al., 2014a,b, 2015; Yeamin et al., 2014). It is worth mentioning that this aspect of the method is a pivotal element for our strategy of developing in order to provide rich and diverse data sets of molecular properties as a cloud service.

### 2.1. The Representation of the PES

**Figure 1:**
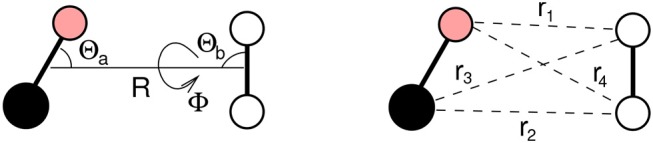
CO-N_2_ dimer with the *R*, Θ_*a*_, Θ_*b*_, Φ coordinates, defining the distance between the molecules and the mutual orientation, and the *r*_*i*_ coordinates defining the distance for all atom-atom interacting pairs.

The intermolecular potential energy, *V*_*inter*_, of the two interacting molecules, CO and N_2_, is formulated as a combination of two effective interaction components:

(1) $$\begin{array}{l} V_{inter}=V_{vdW}+V_{elect}. \end{array}$$

where *V*_*vdW*_ and *V*_*elect*_ represent the van der Waals (size repulsion plus dispersion-attraction) and the electrostatic interaction components, respectively. *V*_*elect*_ originates from the anisotropic molecular charge distributions of the two bodies, which asymptotically tend to the sum of the (permanent) quadrupole-(permanent) quadrupole and dipole-quadrupole interactions. Both *V*_*vdW*_ and *V*_*elect*_ depend on the distance *R* between the centers of mass of the two molecules (say *a* for CO and *b* for N_2_), and on the Jacobi angular coordinates Θ_*a*_, Θ_*b*_ and Φ describing the *a* − *b* mutual orientation as well (see Figure 1). In the present work we will consider a series of limiting configurations of the interacting molecules, specifically (Θ_*a*_,Θ_*b*_,Φ)=(90°, 90°, 0°), (90°, 90°, 90°), (90°, 0°, 0°), (0°, 90°, 0°), (180°, 90°, 0°), (0°, 0°, 0°) and (180°, 0°, 0°), which will be referred to as H, X, T_*a*_, T_*b*1_, T_*b*2_, I_1_ and I_2_.

The van der Waals term, *V*_*vdW*_ of Equation (1), is expressed as a sum of the noncovalent contributions $V_{vdW}^{i}$ as follows,

(2) $$\begin{array}{l} V_{vdW}\left(R\right)=\overset{4}{\underset{i=1}{\sum }}V_{vdW}^{i}\left(r_{i}\right), \end{array}$$

where *r*_*i*_ is the distance between different interaction centers of the involved molecules, which here are chosen to coincide with the constituting atoms (see Figure 1). Accordingly, the sum of Equation (2) runs over all four atom pairs of the CO-N_2_ complex (see Figure 1). It must be emphasized here that in the present work the bond-bond formulation is replaced by the pseudoatom-pseudoatom one in order to properly account for the variation of the anisotropy with the atom-atom distance and the polarizability consistent with the diatom (Pirani et al., 2019).

The explicit form of $V_{vdW}^{i}$ is obtained using the Improved Lennard-Jones (ILJ) potential function (Pirani et al., 2008):

(3) $$\begin{array}{l} \frac{V_{vdW}^{i}\left(r_{i},\right)}{\varepsilon }=f\left(x_{i}\right)=\left[\frac{6}{n\left(x_{i}\right)-6}{\left(\frac{1}{x_{i}}\right)}^{n\left(x_{i}\right)}-\frac{n\left(x_{i}\right)}{n\left(x_{i}\right)-6}{\left(\frac{1}{x_{i}}\right)}^{6}\right] \end{array}$$

where *x*_*i*_ is the reduced distance defined as

(4) $$\begin{array}{l} x_{i}=\frac{r_{i}}{R_{m}}. \end{array}$$

and ε and *R*_*m*_ are, respectively, the well depth and position of the corresponding pair interaction. Note that the ILJ function (Pirani et al., 2008) is more realistic than the original Lennard-Jones (12,6) one, with a much more accurate size repulsion (first term within the square brackets) and the long range dispersion attraction tail (second term within the square brackets) (Pirani et al., 2004; Lombardi and Palazzetti, 2008).

The exponent *n* in Equation (3) is expressed as a function of *x*_*i*_ using the following empirical equation (Pirani et al., 2004):

(5) $$\begin{array}{l} n\left(x_{i}\right)=\beta +4.0x_{i}^{2}. \end{array}$$

in which β is a parameter depending on the nature and the hardness of the interacting particles. For the present system, β has been set equal to 8 (a value typical of van der Waals interactions in neutral-neutral systems) for all atom-atom pairs.

The relevant ε and *R*_*m*_ parameters are directly related to the molecular polarizability of the involved partners, and a zeroth-order estimate of them can be obtained from correlation formulas (Pirani et al., 2001, 2019; Cappelletti et al., 2008) which exploit “effective” atomic polarizability.

In this way a tentative full dimensional PES is generated and related ε and *R*_*m*_ parameters can be fine-tuned by fitting experimental data and by comparing model predictions with accurate ab initio electronic structure calculations (see below).

The *V*_*elect*_ term of Equation (1) has been formulated as a sum of Coulomb potentials as follows:

(6) $$\begin{array}{l} V_{elect}\left(R,\Theta_{a},\Theta_{b},\Phi \right)=\underset{jk}{\sum }\frac{q_{ja}q_{kb}}{r_{jk}} \end{array}$$

with *q*_*ja*_ and *q*_*kb*_ being point charges (located on the monomers *a* and *b*, respectively, and having values consistent with the corresponding calculated molecular dipole and quadrupoles) and *r*_*jk*_ being the distance between them.

Such a formulation of *V*_*elect*_ must be used for cases in which the molecular dimensions are not negligible with respect to the intermolecular distance *R* (Maitland et al., 1987). For both monomers a linear distribution of charges as that used in a previous work, see Lombardi et al. (2016b), has been adopted, which consists of three charges placed on the atoms and on the molecule center of mass.

For charge values *q*_*i*_ (as well as for the corresponding position *r*_*i*_), we took for N_2_ the values reported in Lombardi et al. (2016b) while for CO we obtained them by exploiting corresponding dipole and quadrupole moment calculations (see below) together with simple geometrical considerations (see Appendix A). A comparison of the van der Waals and electrostatic interaction contributions to the intermolecular potential energy, for the various configurations of the two interacting molecules is reported in Figure S1 of the Supplementary Material.

### 2.2. The Optimization of the PES

The above-mentioned adopted values of ε and *R*_*m*_ (hereinafter called “predicted”) were fine-tuned by carrying out a comparison with ab-initio estimates of the intermolecular part of the interaction and an analysis of the second virial coefficient data (see next section). Predicted and optimized values for the case of rigid monomers are given in Table 1 together with the other parameters used to formulate the intermolecular potential.

**Table 1:** Optimized *R*_*m*_ (Å) and ε (meV) parameters and (within parentheses) predicted values estimated for rigid momomers at the equilibrium bond length *r*_*eq*_ (Å) and by considering atomic “effective” polarizabilities α_*x*_ values (Å^3^).

**atom-atom**	**R**_***m***_	**ε**_***i***_	**β**
C–N	3.855 (3.806)	3.788 (4.387)	8
O–N	3.666 (3.626)	3.338 (3.805)	8
	**r**_***eq***_	**α**_***C***_	**α**_***O***_
CO	1.1283^*a*^	1.3592^*b*^	0.6695^*b*^
	**r**_***eq***_	**α**_***N***_	**α**_***N***_
N_2_	1.1007^*c*^	0.8981 ^*d*^	0.8981 ^*d*^
μ (CO) *Q* (CO)	*q*_*C*_ (r_*C*_)	*q*_*O*_ (r_*O*_)	*q*_*cm*_ (r_*cm*_)
0.045^*b*^ -1.456^*b*^	-0.5762^*b*^ (0.6447^*b*^)	-0.7195^*b*^ (-0.4836^*b*^)	1.2957^*b*^ (0.0^*b*^)
*Q* (N_2_)		*q*_*N*_ (r_*N*_)	*q*_*cm*_ (r_*cm*_)
-1.115^*c*^		-0.5154^*e*^ (±0.5505^*e*^)	1.0308^*e*^ (0.0^*e*^)

a *From Linstrom and Mallard (2018)*.

b *present work*.

c *From Bartolomei et al. (2011)*.

d *From Appendix B of Cappelletti et al. (2008)*.

e *From Lombardi et al. (2016b)*.

**Figure 2:**
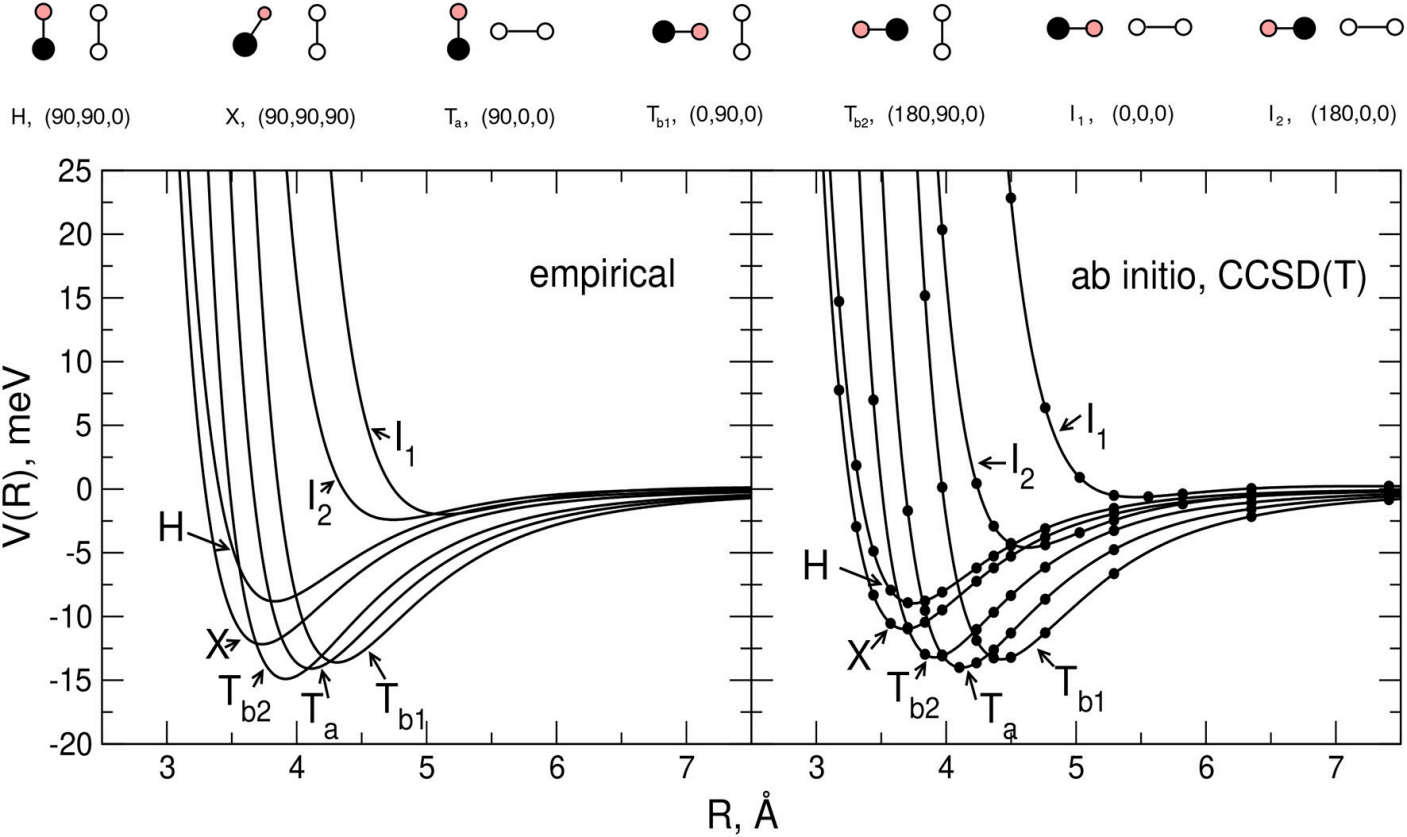
Comparison between the empirical and ab-initio PES for the selected H,X,T_*a*_,T_*b*1_,T_*b*2_,I_1_, and I_2_ configurations of the CO-N_2_ system (rigid monomers). For each reported configuration the C, O, and N atoms are depicted as pink, black and white circles, respectively, and (Θ_*a*_,Θ_*b*_,Φ) represent the Jacobi angular coordinates in degrees. Left hand side panel: present model empirical PES. Right hand side panel: present ab initio calculations (see section 2.2).

In Figure 2 the main features of the intermolecular potential *V*_*inter*_ calculated for seven selected geometries of the interacting system at the CCSD(T) level of theory (see next section) are reported. Although such results will be discussed in more detail later, it is worth pointing out here that, as shown by the figure, the stability ranking of the investigated geometries on the empirical PES model globally agrees well with that of the ab initio values.

In order to carry out the fine tuning of the parameters of the semiempirical functional representation of the PES, we followed the procedure already employed in Bartolomei et al. (2012); Lombardi et al. (2016b), and we first investigated the dimer formation for two rigid monomers (with bond lengths and angles fixed at their equilibrium values). In this perspective, we performed CCSD(T) intermolecular potential calculations for the H, X, T_*a*_, T_*b*1_, T_*b*2_, I_1_, and I_2_ configurations of the dimer (see section 2.1), as a function of the distance *R* of the two monomers. Results are reported in Figure 2 where they are compared with the corresponding empirical PES energy profiles. The supermolecular CCSD(T) energies were calculated using the MOLPRO package (Werner et al., 2006) and corrected using the counterpoise method (Boys and Bernardi, 1970; van Lenthe et al., 1987) in order to remove the basis set superposition error. For all supermolecular calculations, the Dunning's aug-cc-pVTZ basis set (Kendall et al., 1992) was used together with the bond function set [3s3p2d1f] developed by Tao Tao and Pan (1992) and placed on the midpoint of the intermolecular distance *R* that was varied in the range 2.5–8 Å. In order to assess the convergence of the supermolecular energies with the basis set, additional calculations using the aug-cc-pVQZ plus bond functions (see above) basis set were also performed for the usual dimer configurations around their minima. The corresponding results, compared in Table 2 with those obtained using the less extended basis set, show deviations of about 0.1–0.2 meV. The C-O and N_2_ bond lengths of the linear monomers were set equal to 1.1283 Å (Linstrom and Mallard, 2018) and 1.1007 Å (Bartolomei et al., 2011), respectively.

**Table 2:** Equilibrium distance (*R*_*e*_) and binding energy (*D*_*e*_) for the present rigid monomers empirical and ab initio PESs considering selected geometries of the CO-N_2_ dimer (see Figure 2).

		***R***_***e***_ (Å)	***D***_***e***_ (meV)
Empirical	H	3.83 (3.77)	8.81 (10.55)
	X	3.73 (3.68)	11.18 (14.18)
	T_*a*_	4.11 (4.07)	14.09 (15.71)
	T_*b*1_	4.31 (4.27)	13.60 (15.24)
	T_*b*2_	3.91 (3.88)	14.89 (16.51)
	I_1_	5.13 (5.06)	1.99 (2.68)
	I_2_	4.74 (4.68)	2.41 (3.08)
Ab initio	H	3.70 (3.70)	8.92 (9.03)
	X	3.70 (3.70)	10.97 (11.03)
	T_*a*_	4.10 (4.10)	14.00 (14.01)
	T_*b*1_	4.37 (4.37)	13.25 (13.20)
	T_*b*2_	3.97 (3.97)	13.10 (13.08)
	I_1_	5.56 (5.56)	0.60 (0.61)
	I_2_	4.76 (4.76)	4.37 (4.28)

*For the model empirical PES the reported values correspond to those obtained with the optimized and predicted (in parenthesis) R_m_ and ε parameters of Table 1. For the ab initio PES the reported values correspond to those obtained with two different basis sets (see text): aug-cc-pVTZ plus bond functions and aug-cc-pVQZ plus bond functions (in parentheses)*.

In order to carry out a comparison with the flexible monomer empirical potential (to be described below), further CCSD(T) calculations were carried out by considering a maximum variation of 10% in the bond length of one of the two monomers. Such small deformations did not alter the single determinant character (Lee and Taylor, 1989) of the used wavefunctions and allowed the use of the CCSD(T) method also for the deformed arrangements. The ab initio interaction profiles related to flexible monomers, reported in the following, have been compared with the results of the model potential in **Figures 6**, **7**, to prove the validity of the model predictions.

In order to introduce into the empirical model potential the dependence of the electrostatic component *V*_*elect*_ (see Equation 1) on monomer deformations, the variation of the CO and N_2_ molecular multipole moments with the internal coordinates (and consequently, also that of the related charge distributions) was introduced. As for N_2_, the molecular multipoles and the related point charge dependence as those adopted in Lombardi et al. (2016b) were used. In the case of CO, as previously done for CO_2_ (Bartolomei et al., 2012) and N_2_ (Lombardi et al., 2016b), multireference ACPF (Averaged Coupled Pair Functional) calculations were performed as a function of the stretching for the molecular dipole and quadrupole moments by following the guidelines reported in Bartolomei et al. (2011). In particular, the molecular orbitals in the ACPF calculations were all taken to be these natural ones from the complete active space self-consistent field (CASSCF) reference wave functions. Therefore, the considered active space (CAS) was assumed to distribute 10 electrons in the orbitals indicated as (2, 3, 4)σ_*g*_(2, 3, 4)σ_*u*_(1, 2)π_*u*_(1, 2)π_*g*_. The 1σ_*g*_1σ_*u*_ core molecular orbitals were fully optimized, while being constrained to be doubly occupied and excluded from the used CAS. The Dunning's aug-cc-pV5Z basis set (Kendall et al., 1992) was employed and the calculations were performed using the MOLPRO package (Werner et al., 2006).

The parameters of the rigid monomers defining the empirical PES were optimized in order to best fit at the same time the measured second virial coefficients and the ab initio interaction energies. It is worth pointing out here that, given the physical ground of the procedure, both the number of parameters allowed to vary in the fit and their interval of variation are rather small. As an example, the long-range dispersion attraction coefficient values, defined as $\varepsilon \cdot R_{m}^{6}$ were allowed to vary within a 10% interval of their initial value (see also Appendix A of Cappelletti et al., 2008). Moreover, it is also worth pointing out here that, due to some inter-dependencies, the final best fit values of the adjustable parameters usually differ only by a few percent from the initial ones (see Table 1 for ε and *R*_*m*_). The main features (equilibrium distance *R*_*e*_ and binding energy *D*_*e*_ for selected dimer geometries) of the optimized and predicted empirical PES's are reported in Table 2, and it can be seen that the small variations of the optimized ϵ and *R*_*m*_ (see Table 1) lead in general to a less attractive potential. Table 2 shows also that while optimized and predicted parameter values are both able to satisfactorily reproduce the relative stability of the limiting configurations (see Table 2), the former are closer to ab initio results, especially for the most attractive dimer geometries. However, it can also be noticed that the model PES is not capable of predicting the T_*a*_ configuration as the most stable of those here considered; nevertheless, it has to be stressed that the ab initio energy difference between the T_*a*_, T_*b*1_, and T_*b*2_ configurations is quite low and less than 1 meV (see Table 2) and that the very few parameters of the model PES do not allow the description of this very fine behavior.

**Figure 3:**
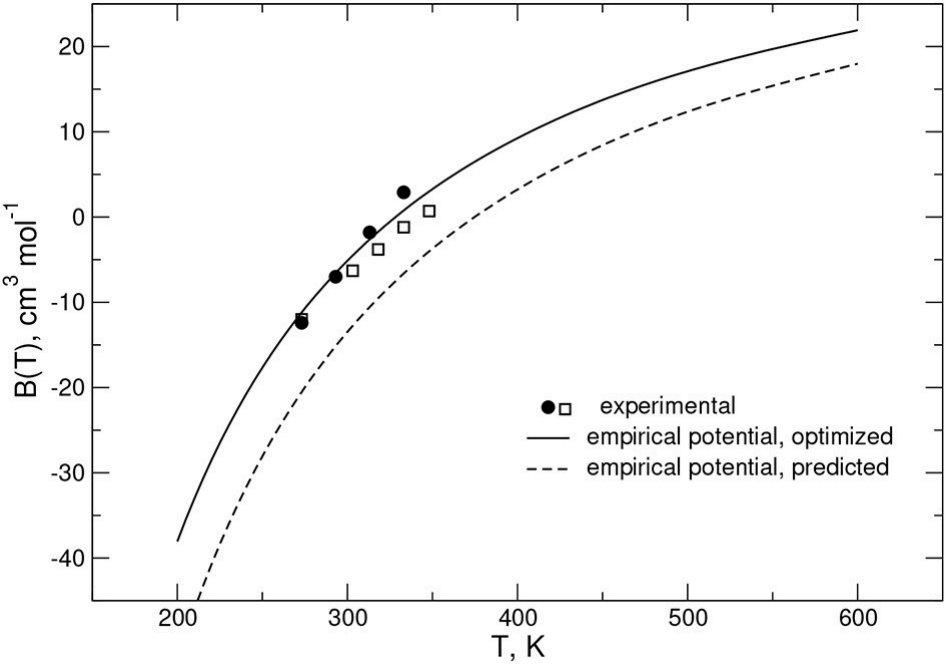
Second virial coefficients for the CO-N_2_ system: solid circles and open squares refer to experimental data from Jaeschke et al. (1988) and McElroy and Buchanan (1995), respectively; calculations obtained from the empirical PES are shown as solid and dashed black lines and correspond to the optimized and predicted potentials, respectively (see Table 2).

As anticipated above, to validate and optimize the rigid rotor PES, second virial coefficient (*B*(*T*)) values, including first quantum correction *B*_*q*1_(*T*) to the classical estimate *B*_*cl*_(*T*) (Pack, 1978) (*B*(*T*) = *B*_*cl*_(*T*)+*B*_*q*1_(*T*)), were also computed. The calculations evidence that quantum corrections are smaller than 1% even for temperature values as low as 200 K. A comparison of calculated *B*(*T*) values with experimental measurements (Jaeschke et al., 1988; McElroy and Buchanan, 1995) over the temperature range 273 < T < 350 K is given in Figure 3.

The figure shows that the predicted PES results underestimate the experimental data in the considered range of temperature, while those referring to the optimized PES lie between the two sets of the measured values. This behavior suggests that, in agreement with the above reported analysis for the ab initio energy values, the predicted PES provides an interaction that is too attractive, which indeed can be properly corrected by tuning the involved ε and *R*_*m*_ parameters.

**Figure 4:**
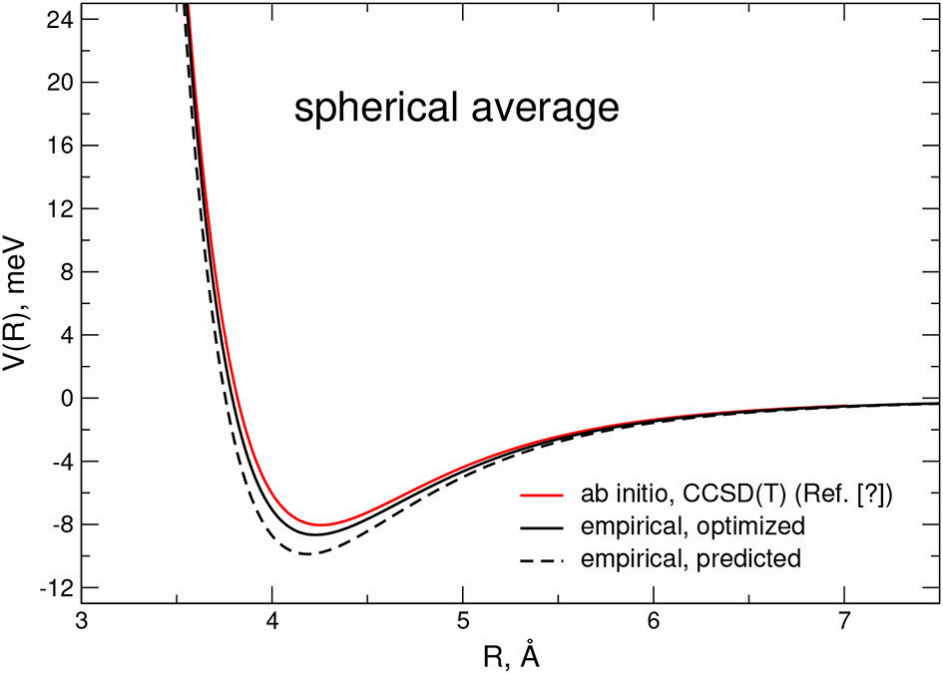
Spherical averaged interaction potential for the CO-N_2_ dimer as obtained from ab initio computations (Surin et al., 2018) at the CCSD(T) level of theory and from the present empirical PES. Estimations from the the optimized and predicted potentials are depicted as black solid and dashed lines, respectively.

A further check of the reliability of the empirical PES is reported in Figure 4, where the related spherical averaged potential is compared with an accurate ab initio estimate at the MP4 level and obtained from Karimi-Jafari et al. (2011). Again, it has to be pointed out that although the predicted PES shows a 15% deeper well, it becomes practically negligible after optimization.

### 2.3. Extension of the PES to Consider Flexible Monomers

The parametrization of the empirical PES has been generalized by properly introducing the dependence of *V*_*vdW*_ and *V*_*elec*_ (see Equation 1) on the molecular elongation. Specifically, the variation of the electric multipole affecting *V*_*elec*_ has been obtained as indicated above, while the change of the molecular polarizability has been taken into account for the modulation of the *V*_*vdW*_ potential parameters.

For the N_2_ monomer deformation, the empirical bond length dependence of the molecular polarizability α as reported in Appendix B of Cappelletti et al. (2008) is used, while for the corresponding point charges distribution dependence the analytic formulas given in Appendix A of Lombardi et al. (2016b) are employed.

**Figure 5:**
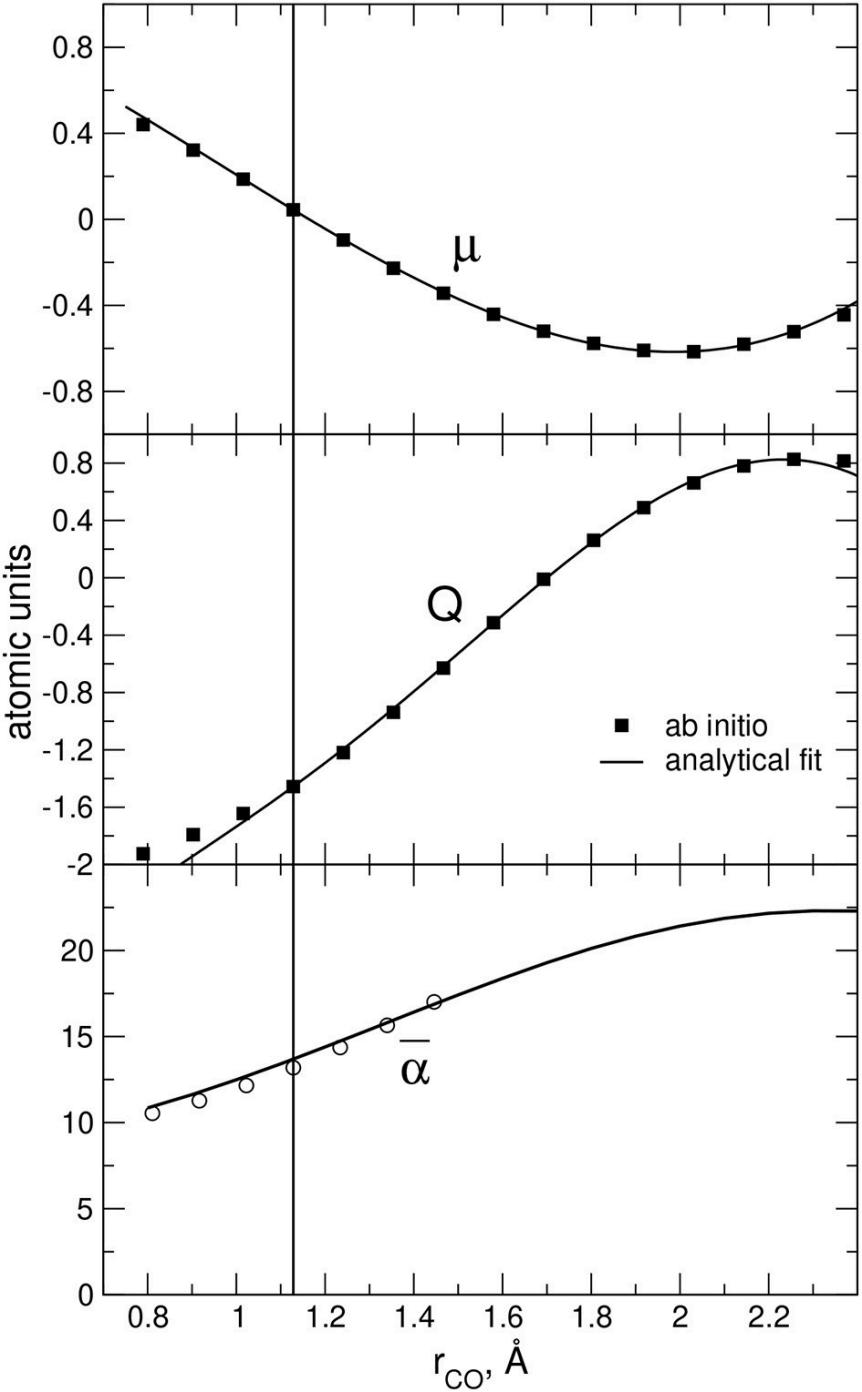
CO electric dipole (μ) and quadrupole (*Q*) moments as well as molecular polarizability ($\bar{\alpha }$) plotted as a function of the interatomic distance *r*. Full squares refer to present ab initio values obtained as detailed in the text, while solid lines correspond to analytic fits (see text and Appendix A). Open circles in the lowest panel refer to previous ab initio estimations at the CCSD(T) level from Maroulis (1996). The vertical line indicates the CO equilibrium distance.

In the case of the CO monomer we used the empirical bond length dependence of the polarizablity α as reported in Appendix A, which is shown here in the lower panel of Figure 5 together with previous ab initio estimates. In addition, for the CO permanent dipole quadrupole dependence on the bond length, an appropriate representation can be obtained from the analysis of present ab initio results reported in the upper panels of Figure 5. To this end, ab initio data were fitted using suitable analytic functions providing the radial dependence of point charges on the CO bond length *r*, as shown in detail in Appendix A.

**Figure 6:**
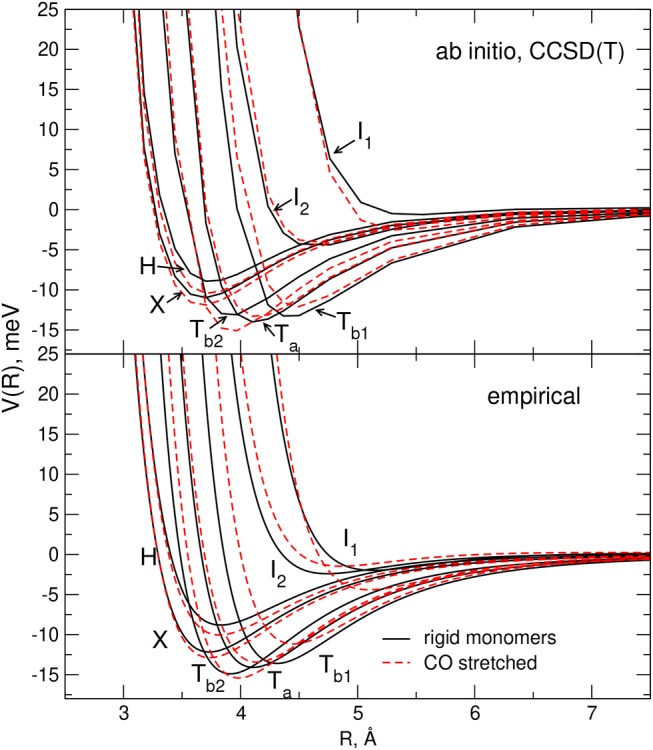
Comparison of the interaction energies for selected dimer geometries of a pair of rigid monomers (black lines) and of a rigid N_2_ plus a stretched CO (the bond length has been elongated of 10% with respect to equilibrium) monomer (red dashed lines). Empirical PES results are plotted in the lower panel while those corresponding to the ab initio calculations are plotted in the upper panel.

**Figure 7:**
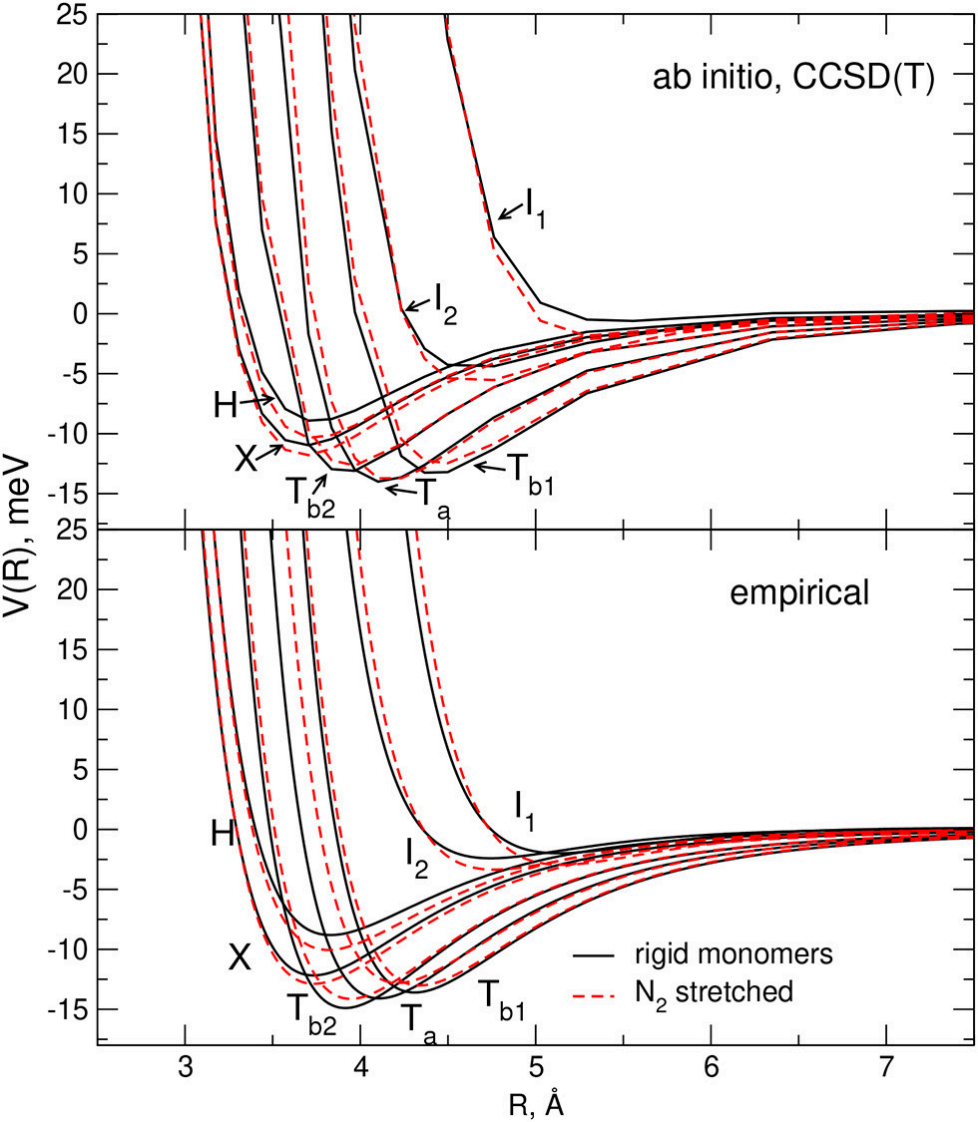
Comparison of the interaction energies for selected dimer geometries of a pair of rigid monomers (black lines) and of a rigid CO plus a stretched N_2_ (the bond length has been elongated of 10% with respect to equilibrium) monomer (red dashed lines). Empirical PES results are plotted in the lower panel while those corresponding to the ab initio calculations are plotted in the upper panel.

The effect of the stretching of a single monomer on the complex interaction predicted by the adopted potential formulation is illustrated in Figures 6, 7. In all cases the plots of the CCSD(T) values, obtained for rigid and flexible molecules, are also reported for comparison: it can be appreciated that the elongation of one monomer provokes the same trends in both PESs for the stability of the considered interaction profiles, confirming therefore the reliability of the adopted empirical model.

## 3. Quantum Classical Calculations for Vibrational Energy Transfer Processes

As a premise to this section, we remark that, when collisions involve more than three atoms, it is impractical to ground realistic simulations and the related systematic computations on full dimensional quantum methods, solving the associated Schrödinger equations, since their efficiency is strongly dependent by the number of degrees of freedom. Although work is constantly being done to achieve the feasibility of exact quantum calculations (e.g., through new coordinates and basis sets Aquilanti et al., 2000, 2002, 2004a,b, 2006; Sevyuk et al., 2005; Castro Palacio et al., 2007; Barreto et al., 2011, 2012; Palazzetti et al., 2011), these remain mainly limited to three-atom systems. For energy transfer, reactive and photodissociating systems involving triatomic and larger molecules, or for the simulation of more complex environments such as gaseous mixtures and flows, classical trajectories are widely employed to interpret experimental results, saving computing time (see e.g., Lombardi et al., 2010; Aquilanti et al., 2011; Palazzetti et al., 2013; Nakamura et al., 2015).

Semiclassical methods are also available (Laganà et al., 2003; Faginas-Lago and Laganá, 2005; Lago et al., 2005; Faginas-Lago et al., 2006, 2010; Faginas et al., 2008; Rampino et al., 2012b), but these won't be considered here.

On the other hand, an efficient and accurate method for calculating cross sections and rate coefficients for the exchange of vibrational quanta of energy upon inelastic collisions for the process:

$$\begin{array}{l} CO\left(v_{i}\right)+N_{2}\left(v_{i}^{\prime }\right)\to CO\left(v_{f}\right)+N_{2}\left(v_{f}^{\prime }\right), \end{array}$$

is the Quantum Classical (QC) one introduced and developed by G.D. Billing (see Billing, 1984a, 1987) by combining quantum mechanics treatments (for selected bound degrees of freedom) with classical mechanics ones (for the remainder).

The QC method is still one of the most efficient tools to calculate large numbers of accurate rate coefficients for processes involving vibrational energy transfer. The detailed description of the method goes beyond the aim of the present work. The formulation used here is essentially the one described in detail in Coletti and Billing (1999), Coletti and Billing (2000), Coletti and Billing (2002), Billing et al. (2003), and Fioccola et al. (2017), to which the reader is referred for the relevant mathematical derivation. In this paper we give only the formulation of the basic properties.

### 3.1. The Main Features of the Adopted Quantum Classical Method

According to the QC approach, the quantum mechanical time-dependent Schrödinger equation for the nuclear motion is solved for the degrees of freedom of the system playing the most relevant role in vibration-to-vibration (VV) quantum energy exchange, vibrations and roto-vibrational couplings, by a coupled equations method to obtain the quantum transition amplitudes $a_{vv^{\prime }}\left(t\right)$, where *v* and *v*′ are the initial vibrational quantum numbers of the diatoms. The vibrational wavefunction is initialized as the product of the Morse functions $\phi_{v}^{0}\left(r_{\text{CO}}\right)\phi_{v^{\prime }}^{0}\left(r_{{\text{N}}_{2}}\right)$ for the two infinitely separated diatoms, whose parameters are given in Table 3, and is expanded as:

(7) $$\begin{array}{l} \text{Ψ}=\underset{v,v^{\prime }}{\sum }a_{vv^{\prime }}\left(t\right)\phi_{v}\left(r_{CO}\right)\phi_{v^{\prime }}\left(r_{N_{2}}\right)\exp\left[-\text{i}\hbar^{-1}\left(E_{v}-E_{v^{\prime }}\right)t\right] \end{array}$$

where *E*_*v*_ and $E_{v^{\prime }}$ are the vibrational energies of the oscillators in their initial states. The remaining degrees of freedom are treated classically by integrating the corresponding set of Hamilton equations of motion in an effective potential defined as the Ehrenfest average of the interaction potential.

**Table 3:** Morse and vibrational parameters for CO and N_2_.

	**CO**	**N_**2**_**
ω_*e*_	2169.81 cm^−1^	2359.60 cm^−1^
*x*_*e*_	0.006125	0.006126
*y*_*e*_	0.0000048	0.0000032
$\bar{r}$	1.128 Å	1.098 Å
β	2.298	2.689

The quantum transition amplitudes $a_{v_{f}v_{f}^{\prime }}\left(t\right)$ can then be used to calculate either specific state-to-state vibrational/rotational transitions cross sections, $\sigma_{v_{i}j_{i}v_{i}^{\prime }j_{i}^{\prime }\to v_{f}j_{f}v_{f}^{\prime }j_{f}^{\prime }}$, or Monte Carlo averaged cross sections over the Boltzmann distribution of the initial rotational angular momenta *j* and *j*′ for CO and N_2_, respectively:

(8) $$\begin{array}{l} \sigma_{v,v^{\prime }}\left(U,T_{0}\right)=\frac{\pi \hbar^{6}}{8\mu {\left(kT_{0}\right)}^{3}I_{CO}I_{N_{2}}}\times \\ \int_{0}^{l_{max}}\int_{0}^{j_{max}}\int_{0}^{j^{\prime }max}\text{d}j\text{ d}j^{\prime }\text{ d}l\;\left(2j+1\right)\left(2j^{\prime }+1\right)\\ \left(2l+1\right)N^{-1}\sum |a_{vv^{\prime }}{|}^{2} \end{array}$$

where *T*_0_ is an arbitrary reference temperature, which cancels out in the formulation of rate constants (Equation 9), *I*_*CO*_ and *I*_*N*_2__ are the moments of inertia of the diatoms, *U* is the classical energy, obtained by subtracting from total energy the vibrational energy of the two diatoms, $U=E-E_{v}-E_{v^{\prime }}$, *j*_*max*_ and $j_{max}^{\prime }$ are the upper limit for the randomly chosen rotational quantum numbers for the diatoms, *l*_*max*_ the upper limit for the angular momentum and μ is the reduced mass of the system.

From such averaged cross sections, rate coefficients for vibrational relaxation $k_{vv^{\prime }}\left(T\right)$ (see e.g., Coletti and Billing, 2002; Billing et al., 2003) can be derived as follows:

(9) $$\begin{array}{l} k_{vv^{\prime }}\left(T\right)={\left(\frac{8kT}{\pi \mu }\right)}^{1/2}{\left(\frac{T_{0}}{T}\right)}^{3}\int_{\epsilon_{min}}^{\infty }\text{d}\left(\frac{\bar{U}}{kT}\right)\;{\text{e}}^{-\bar{U}/kT}\sigma_{v,v^{\prime }}\left(T_{0},\bar{U}\right),\; \end{array}$$

where ϵ_*min*_ = 0 for exothermic and ϵ_*min*_ = Δ*E* for endothermic processes and $\bar{U}$ is the symmetrized classical energy (Billing, 1984b):

(10) $$\begin{array}{l} \bar{U}=U+\frac{1}{2}\Delta E+\frac{\Delta E^{2}}{16U} \end{array}$$

which has been introduced to restore, in an approximate fashion, the quantum mechanical detailed balance principle (Billing, 1984a,b, 1987).

In the present treatment the anharmonic vibrational energy is formulated as:

(11) $$\begin{array}{l} E_{v_{i}}=\hbar \omega_{ei}\left(v_{i}+\frac{1}{2}\right)-\hbar \omega_{ei}x_{ei}{\left(v_{i}+\frac{1}{2}\right)}^{2}+\hbar \omega_{ei}y_{ei}{\left(v_{i}+\frac{1}{2}\right)}^{3} \end{array}$$

where ω_*ei*_ is the wavenumber for the *i*-th oscillator and *x*_*ei*_ and *y*_*ei*_ are the anharmonicity constants (see Table 3 for the values employed in the calculation for N_2_ and CO).

### 3.2. Results and Discussion

QC rate coefficients have been computed for the exothermic exchange of a single vibrational quantum of energy between the first excited vibrational level $v_{i}^{\prime }=1$ of N_2_ (N_2_(1)) and the ground vibrational level *v*_*i*_ = 0 of CO (CO(0))

$$\begin{array}{l} \text{CO}\left(0\right)+{\text{N}}_{2}\left(1\right)\to \text{CO}\left(1\right)+{\text{N}}_{2}\left(0\right)+187.45\text{c}{\text{m}}^{-1} \end{array}$$

and for the endothermic exchange of a single vibrational quantum of energy between the first excited vibrational level *v*_*i*_ = 1 of CO (CO(1)) and the ground vibrational level $v_{i}^{\prime }=0$ of N_2_ (N_2_(0))

$$\begin{array}{l} \text{CO}\left(1\right)+{\text{N}}_{2}\left(0\right)\to \text{CO}\left(0\right)+{\text{N}}_{2}\left(1\right)-187.45\text{c}{\text{m}}^{-1} \end{array}$$

and in the temperature range 80–3,000 K, by running trajectories at 15 initial values of total classical energy comprised between 50 cm^−1^ and 15,000 cm^−1^, with a more frequent sampling driven toward lower energies. For each energy value, 2,000 trajectories were considered, which should ensure an accuracy for rate constants of ~ 20% at lower temperatures and ~ 15% at higher ones.

For each trajectory, the impact parameter was randomly chosen between 0 and 10 Å, and the initial separation between the colliding partners was set equal to 15 Å. In the expansion (7) a total of 36 vibrational states was included, i.e., those consisting of a band of energy Δ*E* up to 14,000 cm^−1^.

**Figure 8:**
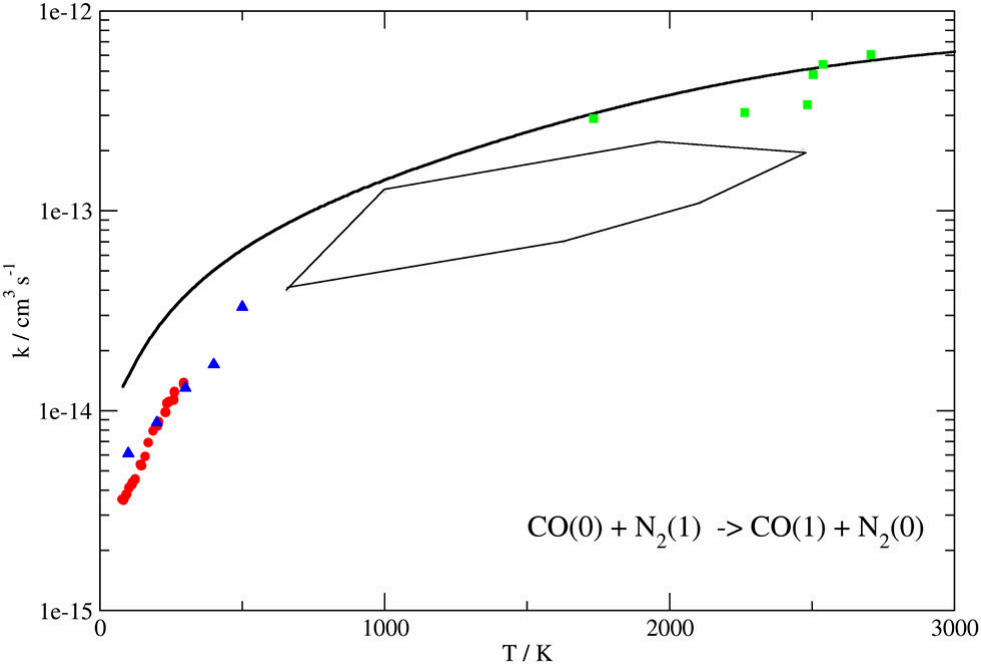
Rate coefficients (logarithmic scale) plotted as a function of temperature for the CO(0)+N_2_(1) → CO(1)+N_2_(0) transition. Present work results (solid line) compared to the experimental ones up to 300 K of Allen and Simpson (1980) (red circles), those obtained by laser-induced fluorescence (blue triangles) (Mastrocinque et al., 1976) and the high temperature ones of Sato et al. (1969) (green squares). The close area represents rate coefficients obtained in von Rosenberg et al. (1972) by shock wave in the range 1,000–2,000 K.

**Figure 9:**
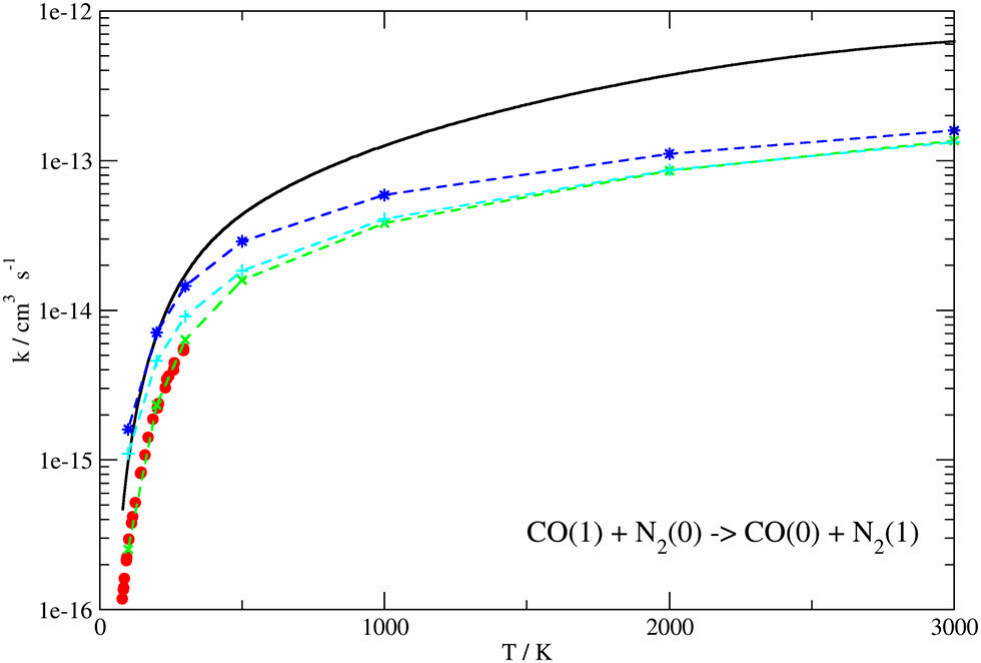
Rate coefficients (logarithmic scale) plotted as a function of temperature for the CO(1)+N_2_(0) → CO(0)+N_2_(1) transition. Present work results (solid line) compared to the experimental ones up to 300 K of Allen and Simpson (1980) (red circles) and those calculated in Kurnosov et al. (2003) using different potentials (dashed lines with points).

Figures 8, 9 show the dependence of the computed QC rate coefficients on the temperature for the exothermic CO(0)+N_2_(1) → CO(1)+N_2_(0) and the endothermic CO(1)+N_2_(0) → CO(0)+N_2_(1) processes, respectively.

For comparison, the same Figures show the experimental data (Sato et al., 1969; von Rosenberg et al., 1972; Mastrocinque et al., 1976; Allen and Simpson, 1980), when available, together with previous theoretical results (Kurnosov et al., 2003).

Rate coefficients computed at some selected temperature values are also reported in Tables 4, 5.

**Table 4:** Experimental and calculated rate constants, in cm^3^ s^−1^, for the exothermic CO(0)+N_2_(1) → CO(1)+N_2_(0)+187.45 cm^−1^ process.

**Temperature**	**This work**	**Experimental Allen and Simpson (1980)**
80	1.27 ·10^−14^	3.60·10^−15^
90	1.40·10^−14^	3.83·10^−15^
100	1.48·10^−14^	4.11·10^−15^
150	1.98·10^−14^	5.90·10^−15^
200	2.51·10^−14^	8.62·10^−15^
250	3.10·10^−14^	1.20·10^−14^
300	3.65·10^−14^	1.40·10^−14^
500	6.19·10^−14^	
1,000	1.39·10^−13^	
1,500	2.46·10^−13^	
2,000	3.78·10^−13^	
2,500	5.14·10^−13^	
3,000	6.22·10^−13^	

**Table 5:** Experimental and calculated rate constants, in cm^3^ s^−1^, for the endothermic CO(1)+N_2_(0) → CO(0)+N_2_(1)-187.45 cm^−1^ process.

**Temperature**	**This work**	**Expt. Allen and Simpson (1980)**	**Calc. Kurnosov et al. (2003) pot I**	**Calc. Kurnosov et al. (2003) pot II**	**Calc. Kurnosov et al. (2003) pot III**
80	4.62 ·10^−16^	1.36·10^−16^			
90	7.00·10^−16^	2.13·10^−16^			
100	9.90·10^−16^	2.95·10^−16^	2.53·10^−16^	1.10·10^−15^	1.60·10^−15^
150	2.98·10^−15^	1.08·10^−15^			
200	6.46·10^−15^	2.22·10^−15^	2.33·10^−15^	4.60·10^−15^	7.10·10^−15^
250	1.06·10^−14^	4.01·10^−15^			
300	1.25·10^−14^	5.52·10^−15^	6.35·10^−15^	9.13·10^−15^	1.45·10^−14^
500	4.24·10^−14^		1.59·10^−14^	1.84·10^−14^	2.89·10^−14^
1,000	1.24·10^−13^		3.81·10^−14^	4.08·10^−14^	5.89·10^−14^
1,500	2.34·10^−13^			
2,000	3.71·10^−13^		8.55·10^−14^	8.64·10^−14^	1.11 ·10^−13^
2,500	5.12·10^−13^			
3,000	6.25·10^−13^		1.36·10^−13^	1.33·10^−13^	1.59·10^−13^

The behavior of the QC rate coefficient plots computed on our PES shown in Figures 8, 9 for both processes agrees well with the experimental ones over the whole temperature range. More in detail, deviations by a maximum factor of 3 are found at the lowest values of T, where the experimental data are more bound to be affected by lower accuracy, although the corresponding slope is well reproduced. Indeed the calculated and experimental value of β in the Arrhenius expression $k\left(T\right)=A\exp\left(-\frac{\beta }{RT}\right)$ for the T range 80–300 K differs for little less than 10%.

In the case of the exothermic VV exchange, where experimental data are available in a wider range of temperatures up to 3,000 K, the behavior at low temperatures is the same as for the endothermic process, but the deviation between calculated and experimental rate coefficients is found to decrease to ~ 20% at temperatures larger than 1,000 K (Figure 8). Thus, the agreement between theoretical and experimental data is rather good in the whole temperature range. Agreement at higher temperatures (superior than in previous theoretical determination) is an indication that the basic contributions to the interaction are well described. The slightly larger discrepancy at low temperature can be attributed either to the lower accuracy of low-temperature experiments or to the neglecting of a proper quantum treatment for rotations which might play a role in the vibrational energy exchange process at low collision energies.

## 4. The Prototyping of the Open Molecular Science Cloud Service

In this section the present implementation of the above-mentioned MOSEX [MOlecular Simulator Enabled Cloud Services (MOSEX) (Vitillaro and Laganà, 2018)], which aimed to support research in computational simulations of molecular scattering processes, is illustrated with particular focus on related progress in the following:

- a) networking and networked software applications,
- b) assembling cloud computing infrastructures,
- c) developing a sustainable operational model.

### 4.1. Networking and Networked Software Applications

**Figure 10:**
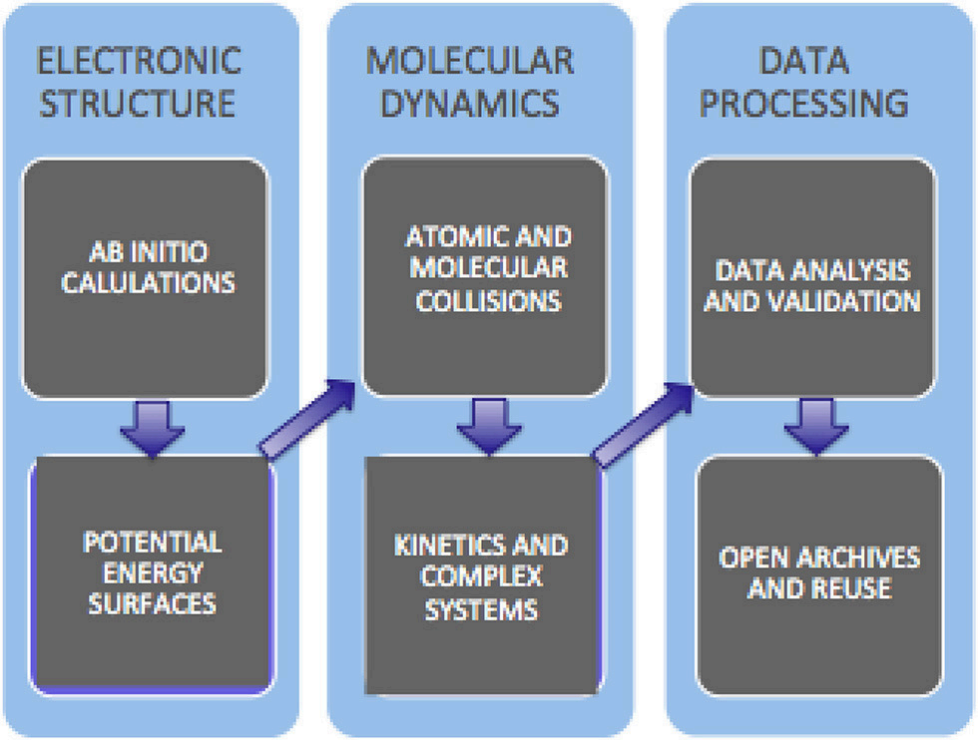
Block diagram of the present evolution of GEMS.

Networking and assembling networked software applications for the Molecular science community begun within COST (www.cost.eu/) Action D23 (METACHEM) and D37 (Grid Computing in Chemistry: GRIDCHEM). In METACHEM the activities of various Molecular Science research laboratories were networked on a shared computing platform made of a geographically distributed cluster of heterogeneous computers operating as a single virtual parallel machine (Foster and Kesselman, 1999). In the following Action GRIDCHEM Grid solutions and paradigms for molecular science research developed by D23 were consolidated on the grid. GRIDCHEM leveraged the creation and the use of distributed computing infrastructures (the “Grid”) to drive collaborative computer modeling and simulation in chemistry toward “new frontiers in complexity and a new regime of time-to-solution” (GRIDCHEM, 2006–2010). At more infrastructural level, the European projects EGEE (Enabling Grids for E-sciencE, https://cordis.europa.eu/project/rcn/87264_en.html), first, and the EGI (European Grid Infrastructure, https://en.wikipedia.org/wiki/European-Grid-Infrastructure), next, provided various disciplines, including Molecular Science, with a world class level platform for computational collaborations. In particular, during EGEE-III, the first pilot computational application called GEMS (Laganà et al., 2010)—designed to enable the distributed calculation of cross sections and rate coefficients starting from the ab initio computation of the electronic structure of the molecular system followed by the fitting of the computed ab initio points using a combination of analytic formulae into a proper functional representation of the interaction in short, intermediate and long-range regions—was implemented. This allows at present routine computation of reactive and nonreactive properties of elementary systems based on the desired number of molecular geometries (Storchi et al., 2006) and collisional paths (Gervasi and Laganà, 2004) by distributing them on the grid. The scheme of the most recent evolution of GEMS toward the atomistic simulation of the kinetics of more complex systems, together with the related “data analysis and validation” and “open archive and reuse” in a cloud service perspective, is sketched in Figure 10.

The molecular science community has been accordingly organized into the COMPCHEM (Laganà et al., 2010) VO first and in the CMMST (Chemistry, Molecular and Materials Science a Technologies) VRC (Virtual Research Community) (https://wiki.egi.eu/wiki/Towards_a_CMMST_VRC) later. On a national basis, then, the different disciplinary communities were gathered in Joint Research Units (JRU), for example in Italy with IGI (http://www.italiangrid.it), in order to give a higher local momentum to networked applications. In this spirit, the following activities were pursued in networked Molecular Sciences:

- orchestrate the initiatives of the e-infrastructure experts and of the disciplinary researchers so as to enable an effective intra- and trans-community networked implementation and coordination of a collaborative/competitive (synergistic) research environment by allowing a selection of compute resources based on quality parameters;
- compose higher level of complexity chained applications through the coordinated usage of distributed hardware and software, the creation of specialized web portals and workflows facilitating the production of data and innovative know how, the direct re-use of the produced data and knowledge in education, training and further research, the rewarding of the work done on behalf of the community by proactive members;
- produce and provide computational services useful to enhancing multi-scale treatments necessary to reproduce the observables of realistic systems in the areas of energy, environment, materials, pharmacology, biology, biotechnologies, medicine, etc., by means of state-of-the-art first principle electronic structure and nuclei dynamics computations, high level of accuracy multiscale design of complex molecular systems, knowledge management for training and education in sciences and technologies;
- turn (in collaboration with partner SMEs), the versatility of the adopted e-infrastructure tools, the richness of the developed CMMST knowledge and the credit mechanism supporting the synergistic operating into a business model, enabling an efficient transfer of the activities to the market, thereby ensuring business sustainability by leveraging synergistic models.

### 4.2. Assembling Cloud Computing Infrastructures

The cloud-oriented computing infrastructure developed for our calculations is an embryonic platform of the Beowulf type running under the OpenStack platform sketched in the lower side of Figure 3 of Vitillaro and Laganà (2018) named HERLA (https://en.wikipedia.org/wiki/Beowulf_cluster). HERLA has been established at the Dipartimento di Chimica, Biologia e Biotecnologia (DCBB) of the University of Perugia. The platform consists of a couple of HPC clusters, (CG/training) and (FE/research) running Scientific Linux 6.x with two distinct access nodes. The clusters are connected using NIS in a single-image system and are used first for students' training (CG) and second for scientists' research (FE). The management of Herla is presently carried out by the CMS^2^ Consortium (http://www.cms-2.org/index.php) of the University of Perugia, CNR-ISTM - Perugia unit and the two companies Master-UP s.r.l. and Molecular Horizon s.r.l. Recently, cloud images of HERLA (VHERLA) have been created and deployed in a storage system (CEPH located at DCBB). This effort was meant to support as well the activities of the School on Open Science Cloud (SOSC17) held in Perugia on June 2017 in collaboration with the Department of Physics and Geology (DFG) and INFN Perugia (running under the INFN OpenStack platform sketched in the lhs upper side of Figure 3 of Vitillaro and Laganà, 2018).

Later, as sketched in the rhs side of Figure 4 of Vitillaro and Laganà (2018), the images of VHERLA were allocated to the OpenStack GARR Cloud platform in Palermo within the project “cnr-istm” and were used to install the version VHERLA(GARR-CLOUD) hscw (http://hscw.herla.unipg.it/ganglia/?p=2&c=FrontEnd) bearing the following features: Access node hscw (2 core, 4Gb RAM, 200Gb storage) and Cluster (7 nodes, 96 cores, 360Gb RAM, 700Gb storage). The Access node hscw, can be reached at the IP address [90.147.189.20] via SSH and the following 7 nodes, 96core, 380Gb, 512Gb scratch are defined at Torque(PBS)/MAUI as [Intel Xeon E3-12xx v2(Ivy Bridge)/2.6Ghz]. This allowed the generation of a virtual cluster for Molecular Sciences that has also been used for the training of the Students of the XIII EM TCCM (European Master in Theoretical Chemistry and Computational Modeling) Intensive Course (http://www-old.chm.unipg.it/chimgen/mb/theo2/TCCM2018/EM- TCCM2018/EM-TCCM/Welcome.html) with details at the following reference web page: http://hscw.herla.unipg.it URL.

### 4.3. Developing a Sustainable Operational Model

As to the operational model we have already developed a sustainable open collaborative user/producer (Prosumer), whose prototype has been first implemented for running the ECTN (European Chemistry Thematic Network (http://ectn.eu/)) EChemTest *e*-tests based on the use of the educational LibreEOL (https://echemtest.libreeol.org) and GLOREP (https://glorep.unipg.it/) services Laganà et al., 2018a. An important feature of the Prosumer model adopted for EChemTest was the containment of costs by leveraging the fact that the ECTN member HEIs running *e*-test Self Evaluation Sessions (SES)s for the assessment of the Chemistry competences of their own students already act at the same time as consumers of EChemTest services and as producers of Question and Answers (Q&A)s, assessors of students' competences, designers and developers of e-learning materials (a typical cluster of the horizontal type) for the harmonization at the European level of the assessment of Molecular Science competences. This behavior is quite usual in education and research activities in which knowledge is a common good to be at the same time produced and consumed.

The only additional action needed to the end of making the prosumer model sustainable was to assign to a company the role of market spinner. For EChemTest this role was taken by Master-Up s.r.l. thanks to its nature as a former spinoff of the University of Perugia (started in the year 2004 out of the aggregation of some members of the Chemistry Department and the Mathematics and Informatics Department who were experts in molecular dynamics simulations and computer science) devoted to design, production and marketing services for technological innovation as its main goal. As a matter of fact, the mission of Master-Up has been since the very beginning the design and development of cloud services for molecular sciences and technologies. In the particular case of EChemTest, the Prosumer model has led to the production in the year 2018 of 2622 SESs, with an increase of 5 percent over the previous year. Further efforts have also been spent in the period up to January 2019 for the implementation of the already mentioned MOlecular Simulator Enabled Cloud Services (MOSEX), a European Open Science Cloud Pilot designed as a follow-up of the EGI COMPCHEM VO activities for the following applications:

1. Molecular electronic structure and dynamical properties programs of GEMS (including the QC ones reported reported in this paper)
2. Drug design programs (QSPR and 3D QSPR models)
3. Distributed repository of molecular science data (including the IOCHEM (https://www.iochem-bd.org/) software for the organization, publication and storage of molecular information on materials)
4. Dissemination and evaluation of molecular knowledge through GLOREP (Distributed repository of shared Learning Objects and educational tools) for the assemblage of Molecular Science multimedia Learning Objects.

## 5. Conclusions

In this work we have presented an accurate full dimensional potential energy surface for the CO-N_2_ system, characterized by an extension of the bond-bond formulation of the intermolecular interactions. The PES, intended for use in dynamics simulations aimed at calculating sets of accurate state-to-state rate coefficients, has been validated by performing Quantum-Classical dynamics simulations of the CO-N_2_ vibrational energy exchange, with outcoming rate coefficients in substantial agreement with experimental data on a wide range of temperatures. The generation of large pools of kinetics data and accurate molecular properties to feed databases employed in astrochemical models, plasma chemistry and combustion studies, is a current challenge for theoretical and computational chemistry, with a strong multidisciplinary character. Here we suggest to set up cloud-oriented computing infrastructures based on collaborative computer modeling and simulations to gather communities and boost efforts.

## Author Contributions

AaL and AoL conceived and supervised the study and wrote the manuscript. FP and MB formulated the PES and performed ab initio calculations. CC performed dynamics simulations based on the Quantum Classical method, obtaining the theoretical rate coefficients.

### Conflict of Interest Statement

The authors declare that the research was conducted in the absence of any commercial or financial relationships that could be construed as a potential conflict of interest.

## Appendix A

### Dependence of the CO Polarizability and Permanent Multipoles on the Monomer Bond Length

The radial dependence of the CO average polarizability $\bar{\alpha }$ (in Å^3^) is defined by the following empirical equations given in terms of *n*, an effective bond order, *r*, the bond length, and *r*_*eq*_ its equilibrium value (see Table 1):

(A1) $$\begin{array}{l} \bar{\alpha }(r)=(0.5733n+0.8400)\{1+\frac{1}{3}\left(\frac{r}{r_{eq}}-1\right)\\ \exp\left[1.0961\left(\frac{r}{r_{eq}}-1\right)\left(1-0.4682r\right)\sqrt{\frac{r}{r_{eq}}}\right]\} \end{array}$$

where the effective bond order reads

(A2) $$\begin{array}{l} n=3-\left(\frac{0.6538\frac{r}{r_{eq}}+1.3076}{1+0.8770r^{2}}\right)\exp\left[-0.9635r\left(\frac{r}{r_{eq}}-1\right)\right] \end{array}$$

The dependence of $\bar{\alpha }$ on *r* is reported in the bottom panel of Figure 5 where a comparison with previous ab initio estimates is also provided.

The atomic polarizabilities related to the C and O atoms are obtained as

$$\begin{array}{l} \alpha_{C}=0.66\bar{\alpha },\alpha_{O}=0.33\bar{\alpha } \end{array}$$

while that for the N atom (α_*N*_) is assumed to be half the N_2_ molecular polarizability which is obtained from the Appendix B of Cappelletti et al. (2008).

The atomic polarizabilities of the involved molecular partners are then used to obtain the *R*_*m*_ and ε interaction parameters for each atom_*a*_-atom pair_*b*_ as follows Pirani et al. (2001):

(A3) $$R_{m}=1.767\frac{\alpha_{\;a}^{\prime \;1/3}+\alpha_{\;b}^{\prime \;1/3}}{{\left(\alpha_{a}\alpha_{b}\right)}^{0.095}}Å$$

with $\alpha_{\;C}^{\prime }$= 1.382 α_*C*_, $\alpha_{\;O}^{\prime }$= 1.382 α_*O*_ and $\alpha_{\;N}^{\prime }$= 1.65 α_*N*_, and

(A4) $$\begin{array}{l} \varepsilon =0.72\frac{C_{disp}}{R_{m}^{6}}\text{meV} \end{array}$$

being

$$C_{disp}=15.7\times 10^{3}\frac{\alpha_{a}\alpha_{b}}{{\left(\alpha_{a}/{\text{N}}_{a}\right)}^{1/2}+{\left(\alpha_{b}/{\text{N}}_{b}\right)}^{1/2}}\text{me}{\text{V}Å}^{6}$$

an efficient dispersion coefficient depending on the numerical coefficients *N*_*C*_, *N*_*O*_ and *N*_*N*_ with the meaning of the total atomic effective electron numbers which assume the 4, 6, and 4.4 values, respectively.

The radial dependence of the CO molecular dipole (μ(*r*)) is formulated as

(A5) $$\begin{array}{l} \mu \left(r\right)=\mu \left(r_{eq}\right)-1.250\left(r-r_{eq}\right)+0.210{\left(r-r_{eq}\right)}^{2}\\ +0.420{\left(r-r_{eq}\right)}^{3}-0.015{\left(r-r_{eq}\right)}^{4} \end{array}$$

where μ(*r*_*eq*_) represents the dipole moment at *r*_*eq*_.

That of the CO molecular quadrupole (*Q*(*r*)) is expressed as

(A6) $$\begin{array}{l} Q\left(r\right)=Q\left(r_{eq}\right)+2.250\left(r-r_{eq}\right)+0.680{\left(r-r_{eq}\right)}^{2}\\ +0.490{\left(r-r_{eq}\right)}^{3}-1.580{\left(r-r_{eq}\right)}^{4}+0.400{\left(r-r_{eq}\right)}^{5} \end{array}$$

being *Q*(*r*_*eq*_) the quadrupole moment at *r*_*eq*_.

Such polynomial representations provide results in atomic units which reproduce the ab initio data illustrated in the top and intermediate panels of Figure 5.

From the above relationships we obtain the molecular charge distribution as

(A7) $$\begin{array}{l} q_{O}=\frac{Q\left(r\right)+\mu \left(r\right)\cdot r_{C}}{r_{C}\cdot r_{O}+r_{O}^{2}}, \end{array}$$

(A8) $$\begin{array}{l} q_{C}=\frac{-\mu \left(r\right)+q_{O}\cdot r_{O}}{r_{C}}, \end{array}$$

and

(A9) $$\begin{array}{l} q_{cm}=-q_{O}-q_{C}, \end{array}$$

where *q*_*O*_, *q*_*C*_, and *q*_*cm*_ are point charges on the oxygen and carbon atoms and CO center of mass (CM), respectively, and *r*_*O*_, *r*_*C*_ are atom positions with respect to CM.
