# Supplementary material for: Full Dimensional Potential Energy Function and Calculation of State-Specific Properties of the CO+N2 Inelastic Processes Within an Open Molecular Science Cloud Perspective
Source: Front Chem. 2019 May 22;7:309. doi: 10.3389/fchem.2019.00309 (PMC6540877; doi:10.3389/fchem.2019.00309)
Supplement: Supplementary file 1 [file Data_Sheet_1.PDF]

SUPPLEMENTARY MATERIAL – COMPARISON OF VAN DER WAALS AND ELECTROSTATIC CONTRIBUTIONS TO THE INTERMOLECULAR POTENTIAL ENERGY

The intermolecular potential in our model is the result of two effective components accounting separately for the van der Waals and the electrostatic interaction contributions, see Eq. 1. The electrostatic interaction contribution is strongly dependent on the mutual orientation of the molecules and is maximum for collinear configurations (when the two molecular axes are aligned on the same line). The Figure 1 below shows the total interaction energy (left hand panel) and the pure electrostatic interaction (right hand panel) for any of the configurations considered in the present work, see Sec. 2.1 and Figs. 2 and 3. It can be seen that the electrostatic interaction deepens the potential wells of about 50 % for collinear configurations, while the contribution for other configurations is less pronounced or, in some case, negligible.

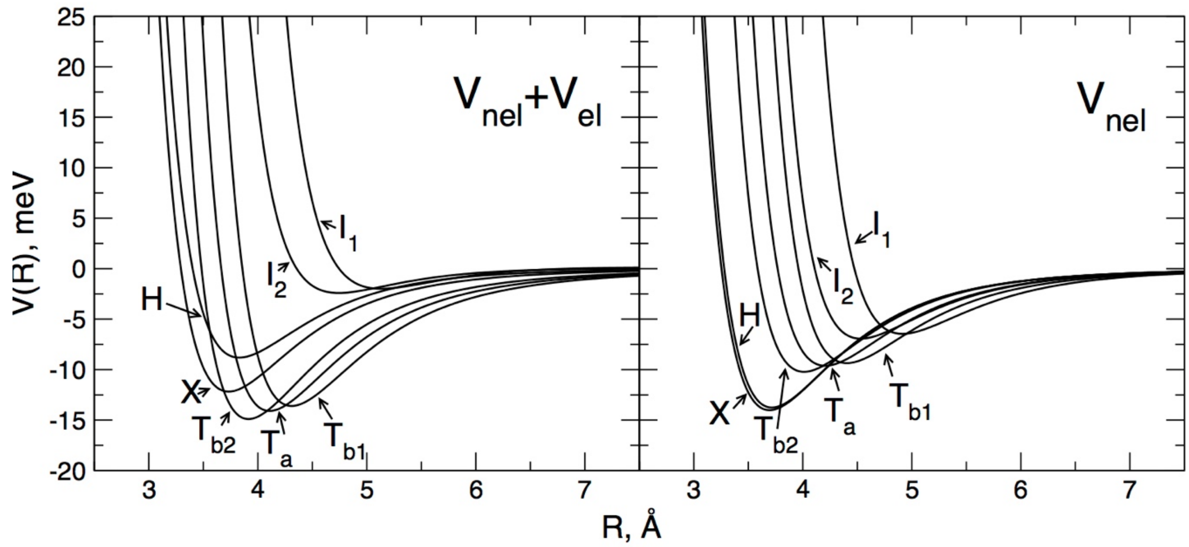

Figure1. Comparison of the van der Waals and electrostatic interaction contributions to the intermolecular potential energy, for the various configurations of the two interacting molecules.
